# Oxidative Stress Induces Nuclear-to-Cytosol Shift of hMSH3, a Potential Mechanism for EMAST in Colorectal Cancer Cells

**DOI:** 10.1371/journal.pone.0050616

**Published:** 2012-11-30

**Authors:** Stephanie S. Tseng-Rogenski, Heekyung Chung, Maike B. Wilk, Shuai Zhang, Moriya Iwaizumi, John M. Carethers

**Affiliations:** 1 Division of Gastroenterology, Department of Internal Medicine, University of Michigan, Ann Arbor, Michigan, United States of America; 2 Department of Medicine, University of California San Diego, San Diego, California, United States of America; Yonsei University College of Medicine, Republic of Korea

## Abstract

**Background:**

Elevated microsatellite alterations at selected tetranucleotide repeats (EMAST) is a genetic signature observed in 60% of sporadic colorectal cancers (CRCs). Unlike microsatellite unstable CRCs where hypermethylation of the DNA mismatch repair (MMR) gene *hMLH1’s* promoter is causal, the precise cause of EMAST is not clearly defined but points towards *hMSH3* deficiency.

**Aim:**

To examine if *hMSH3* deficiency causes EMAST, and to explore mechanisms for its deficiency.

**Methods:**

We measured −4 bp framshifts at *D8S321* and *D20S82* loci within EGFP-containing constructs to determine EMAST formation in MMR-proficient, *hMLH1^−/−^*, *hMSH6^−/−^*, and *hMSH3^−/−^* CRC cells. We observed the subcellular location of hMSH3 with oxidative stress.

**Results:**

*D8S321* mutations occurred 31-and 40-fold higher and *D20S82* mutations occurred 82-and 49-fold higher in *hMLH1^−/−^* and *hMSH3^−/−^* cells, respectively, than in *hMSH6^−/−^* or MMR-proficient cells. *hMSH3* knockdown in MMR-proficient cells caused higher *D8S321* mutation rates (18.14 and 11.14×10^−4^ mutations/cell/generation in two independent clones) than scrambled controls (0 and 0.26×10^−4^ mutations/cell/generation; *p*<0.01). DNA sequencing confirmed the expected frameshift mutations with evidence for ongoing mutations of the constructs. Because EMAST-positive tumors are associated with inflammation, we subjected MMR-proficient cells to oxidative stress via H_2_O_2_ to examine its effect on *hMSH3*. A reversible nuclear-to-cytosol shift of hMSH3 was observed upon H_2_O_2_ treatment.

**Conclusion:**

EMAST is dependent upon the MMR background, with *hMSH3^−/−^* more prone to frameshift mutations than *hMSH6^−/−^*, opposite to frameshift mutations observed for mononucleotide repeats. *hMSH3^−/−^* mimics complete MMR failure (*hMLH1^−/−^*) in inducing EMAST. Given the observed heterogeneous expression of hMSH3 in CRCs with EMAST, *hMSH3*-deficiency appears to be the event that commences EMAST. Oxidative stress, which causes a shift of hMSH3’s subcellular location, may contribute to an hMSH3 loss-of-function phenotype by sequestering it to the cytosol.

## Introduction

DNA mismatch repair (MMR) dysfunction drives the process of microsatellite instability (MSI), observed in nearly all Lynch Syndrome colorectal cancers (CRCs) and in 15–20% of sporadic CRCs [1]. MSI is observed when both alleles of a major DNA MMR gene is inactivated by mutation in Lynch tumors or by hypermethylation in sporadic tumors, causing MMR protein production or function to fail, and allowing frameshifts to occur at DNA microsatellite sequences throughout the genome [1]–[3]. Classic MSI is determined by the occurrence of frameshifts in mono/dinucleotide markers, and has a defined panel for which to compare studies [4]. This panel, or variations of these mono/dinucleotide markers, readily detects *hMLH1*- and *hMSH2*-deficiency (as dysfunction of these two proteins completely inactivates DNA MMR), and occasionally *hMSH6*-deficiency [1], [4]. The MSI panel is not as specific to detect *hMSH3*-deficiency due to the nature of repair that this protein contributes to MMR, based on yeast studies [5], [6]. DNA MMR is an enzyme system whose main cellular function is to repair nucleotide mispairs and insertion/deletion loops (IDLs) during DNA replication. The specificity of mismatch recognition is directed by hMutSα and hMutSβ; hMutSα (hMSH2-hMSH6 heterodimer) binds to single mispairs and small IDLs (≤2 nucleotides), whereas hMutSβ (hMSH2-hMSH3 heterodimer) binds larger IDLs [1], [5]–[7]. We and others have observed DNA lesion binding specificities with purified hMutSα and hMutSβ as well as made specific observations on binding using human CRC cells of differing DNA MMR-deficient backgrounds harboring constructs containing specified-length IDLs or nucleotide mispairs [8]–[12].

Recently, a new form of MSI has been described in up to 60% of sporadic colon [13]–[15] and over 30% of rectal cancers [16]. Unlike classic MSI, elevated microsatellite alterations at selected tetranucleotide repeats (EMAST) is determined by a panel of tetranuleotide markers [13]–[17]. EMAST appears to be more common than classic MSI and seems to be an acquired feature associated with inflammation in CRCs [14], [16]. CRC patients who have EMAST-positive tumors have a poorer prognosis than those with non-EMAST or MSI tumors [16], [18]. EMAST has been observed in various tumors, including non-small cell lung [17], bladder [19], ovarian [20], prostate [21], skin [22], and colorectal [13]–[16], but these studies do not provide full evidence as to the cause of EMAST. In the initial reports of EMAST in colorectal cancers, the authors observed nuclear heterogeneity of hMSH3 within EMAST-positive tumors [13], [14]. Our data, combined with yeast [5], [6], and other human studies [13]–[16], suggests that *hMSH3*-deficiency might be the driver of EMAST in CRCs.

In order to test if *hMSH3*-deficiency is a principal cause of EMAST, we generated tetranucleotide repeat-containing constructs that were fused out-of-frame with EGFP, such that when frameshifted by −4 bp (i.e. deletion of one tetranucleotide unit), EGFP would be expressed. This allowed observation of the generation of EMAST in real-time, and afforded us the ability to calculate mutation frequencies and mutation rates in multiple MMR-deficient backgrounds. In this report, our data clearly shows that *hMSH3*-deficiency is a cause of EMAST in human CRC cells and we further provide a possible mechanism that may contribute to the acquired *hMSH3*-deficiency in human CRCs.

## Results

### Establishment of a Measuring System for EMAST Tetranucleotide Frameshifts

Because some studies have indicated that EMAST is associated with heterogeneous expression of hMSH3 in colorectal tumors [13]–[16], our approach was to generate a system to measure EMAST in a quantitative fashion as a means to directly examine if *hMSH3*-deficiency is the cause of EMAST. Human tetranucleotide *D8S321* and *D20S82* loci sequences (containing 12 and/or 16 repeats of AAAG, respectively) have shown the highest frequency for a −4 bp frameshift in colorectal tumors [14], [16]. These two loci were therefore selected for study using CRC cells with different MMR genetic backgrounds to ascertain subsequent mutation. We have previously established a similar experimental system where deletion of 1 bp of (A)_n_ would lead to the expression of the reporter gene EGFP when a construct was placed +1 bp out-of-frame [10]–[12].

To generate the experimental constructs, we inserted the *D8S321* (D8) and *D20S82* (D20) loci as 60-mers containing the tetranucleotide repeats and surrounding nucleotides immediately after the EGFP start codon [**Table S1**]. Experimental constructs [D8-OF (D8-out-of-frame) and D20-OF] were made +1 bp out-of-frame such that deletion of one AAAG repeat will shift the EGFP reading frame (+1 bp to −3 bp) into the correct frame to cause EGFP expression. Mutation resistant (MR) plasmids (MR-D8 and MR-D20), serving as negative controls, were constructed by replacing the middle AA nucleotides within the AAAG repeats with C/G nucleotides in every third AAAG, interrupting the series of tetranucleotide repeats to prevent frameshift mutations. The MR-D8 and MR-D20 plasmids were placed out-of frame (OF; D8/D20-MR-OF) and/or in-frame (IF; D8/D20-MR-IF) to be used as negative and positive controls for EGFP expression (also see Materials and Methods). These constructs were transfected into human CRC cell lines with different MMR genetic backgrounds and stable cell lines were established via hygromycin B selection. Single cell clones were subsequently established. DNA sequencing was employed to correctly confirm the transfected EMAST constructs (**Table S1**). As expected, cells carrying MR-IF (positive control) were EGFP positive, whereas cells containing MR-OF (negative control) were EGFP negative.

**Figure 1:**
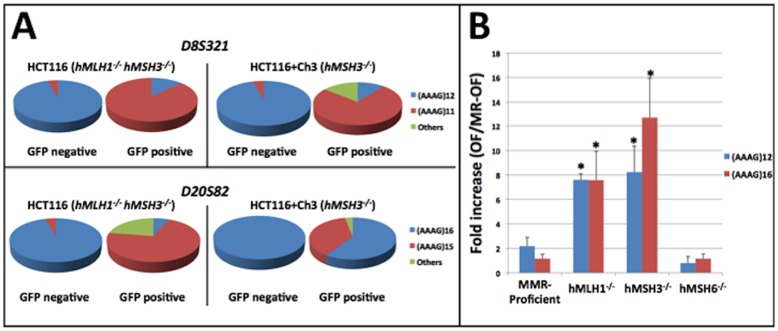
DNA sequencing results and EMAST mutation frequencies in CRC cells with different MMR backgrounds. (**A**) Genomic DNA isolated from EGFP-negative and -positive cells carrying experimental constructs (D8-OF and/or D20-OF) was used to amplify DNA fragments containing EMAST loci to examine if the frameshift mutations had occurred. (**B**) Mutation frequencies were calculated by dividing the percentage of EGFP positive population in the experimental group (OF) by the percentage of EGFP positive in its negative control (MR-OF) to compute the increase of the EGFP-positive population in folds. There were significantly more EGFP-positive cells in *hMLH1*- and *hMSH3*-deficient cells compared to MMR-proficient and/or *hMSH6*-deficient cells. *: p<0.05.

To test if *hMSH3*-deficiency could cause EMAST, non-fluorescent cells carrying the different stably transfected EMAST constructs were sorted by flow cytometry and exponentially grown for four to six weeks. Each week, cells were analyzed in duplicate by flow cytometry for EGFP expression, presumably resulting from deletion of one AAAG repeat from the transfected *D8S321* and/or *D20S82* loci. EGFP positive cells began emerging as soon as one week after EGFP negative cells were sorted and plated (data not shown). To ensure that our experimental system allowed us to correctly assess the occurrence of EMAST, the EGFP negative and positive cells from *hMLH1^−/−^* and/or *hMSH3* ^−/−^ cells harboring the experimental constructs (D8-OF and D20-OF) were sorted to isolate genomic DNA for sequencing. As expected, essentially all clones (greater than 96%) from EGFP negative cells retained wild type (WT) sequences for each of *D8S321* and *D20S82* loci in both *hMLH1^−/−^* and *hMSH3^−/−^* cell lines, whereas clones from EGFP positive cells acquired deletion of one AAAG repeat (**Figure 1A** and **Figure S1A and S1B**). We also observed expansion frameshift mutations at each locus by DNA sequencing (included in “others” in **Figure 1A**). The most frequent one (ranging from 12.5% to as high as 30% of total EGFP-positive cells) was insertion of two AAAG repeats [(AAAG)_12_ to (AAAG)_14_] in *D8S321* that changed the reading frame from +1 bp to +9 bp (listed as “others” in **Figure 1A** and **Figure S1A**). In addition, more than one AAAG repeat deletion (e.g. 4 or 10 AAAG repeat deletion) was rarely observed (<8% of total, listed in “others” in **Figure 1A**). Importantly, deletion/insertion mutations were always detected within the microsatellite sequences while no mutation occurred in the flanking sequences surrounding AAAG repeat (**Figure S1**). These observations indicate that we have established a reliable experimental system to monitor the occurrence of EMAST in CRC cells.

### Frameshift Mutations at Tetranucleotide Repeats of *D8S321* and *D20S82* Loci are Dependent on the MMR Background

To examine the effect of MMR background on frameshift mutations at the tetranucleotide *D8S321* and *D20S82* loci, we calculated mutation frequencies of the markers by comparing each experimental group (OF) to its negative control (MR-OF) using the following formula: “(EGFP positive cells/total live cells from OF)/(EGFP positive cells/total live cells from MR-OF)”. *hMLH1^−/−^* cells (completely devoid of DNA MMR activity) and *hMSH3* ^−/−^ cells showed 7–12 fold higher mutation frequencies compared to the controls for both loci (**Figure 1B**). In particular, the mutation frequencies observed in *hMSH3* ^−/−^ were comparable to that in *hMLH1* ^−/−^ for both loci (**Figure 1B**). The −4 bp mutation rates for both loci in each MMR background were also calculated (**Table 1**). With complete MMR-deficiency (*hMLH1^−/−^*), mutation rates for both loci were 27 and 56×10^−4^ mutation/cell/generation. Similarly, *hMSH3^−/−^* cells generated 33 and 34×10^−4^ mutations/cell/generation. Thus, the mutation rates with *hMSH3*-deficiency are very comparable to those observed for complete MMR-deficiency. This is in sharp contrast to *hMSH6*-deficiency (<1×10^−4^ mutation/cell/generation), essentially identical to MMR-proficient cells. These findings strongly suggest the *hMSH3*-deficiency is the driver for EMAST formation.

**Table 1:** Calculated EMAST mutation rates for *D8S321* and *D20S82* in various MMR backgrounds.

MMR Background	*D8S321* (AAAG)_12_	*D20S82* (AAAG)_16_
MMR-Proficient	2.45×10^−4^±1.04×10^−4^	0.31×10^−4^±0.31×10^−4^
*hMLH1^−/−^*	26.96×10^−4^±1.20×10^−4**a,**^ b	56.4×10^−4^±5.89×10^−4**a,**^ b
*hMSH3^−/−^*	34.82×10^−4^±1.26×10^−4**c,**^ d	33.86×10^−4^±4.25×10^−4**c,**^ d
*hMSH6^−/−^*	0.86×10^−4^±0.86×10^−4^	0.69×10^−4^±0.69×10^−4^

Data from the EGFP-positive population at weeks 4, 5, and 6 time points were used for -4 bp frameshift mutation rate analysis. Single mutation rates were calculated by combining and averaging time-specific mutation rates. Rates are expressed as mutations at microsatellite sequence per cell per generation. Data shown are mean±SEM.

a, b, c, and d represent significant difference in the mutation rate (*P*<0.05).

a *hMLH1^−/−^* vs. MMR-proficient (*D8S321 P* = 0.00012, *D20S82 P* = 0.01069);

b *hMLH1^−/−^* vs. *hMSH6^−/−^* (*D8S321 P* = 0.00012, *D20S82 P* = 0.01021);

c *hMSH3^−/−^* vs. MMR-proficient (*D8S321 P* = 0.00008, *D20S82 P* = 0.01525);

d *hMSH3^−/−^* vs. *hMSH6^−/−^* (*D8S321 P* = 0.0001, *D20S82 P* = 0.01415). Mutations rates were calculated based on the “method of the mean” developed by Luria and Delbruck [25].

### Direct Knockdown of hMSH3 Expression in MMR-proficient Cells Causes EMAST

**Figure 2:**
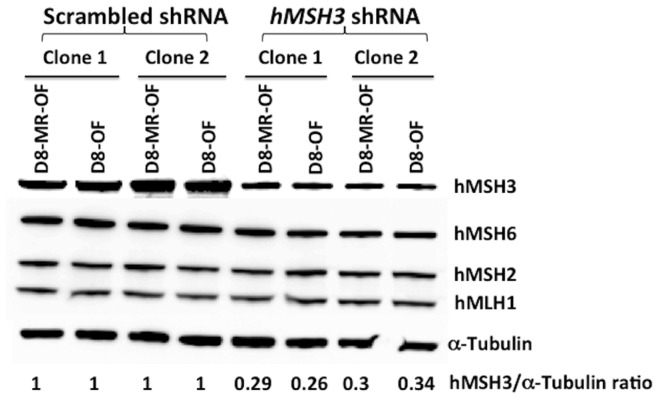
Expression levels of MMR proteins in *hMSH3* knockdown clones. *hMSH3* shRNA transfection successfully and specifically reduced the hMSH3 expression levels in the CRC cell clones. Note that the expression levels of other DNA mismatch repair genes remain unchanged.

To confirm a role of hMSH3 in EMAST, we knocked down *hMSH3* expression in MMR-proficient cells harboring the *D8S321*-EGFP construct by transfecting plasmids carrying *hMSH3*-specific shRNA and/or scrambled control, then measured *D8S321* tetranucleotide frameshift mutations as above. Prior to shRNA transfection, hMSH3 expression levels in chosen MMR-proficient clones were comparable to each other (**Figure S2**). After generation of shRNA stable cell lines (single cell clones), hMSH3 expression was significantly reduced in *hMSH3* shRNA transfected cells (**Figure 2**; varying between 26–34% of the protein levels of the scramble controls).

**Figure 3:**
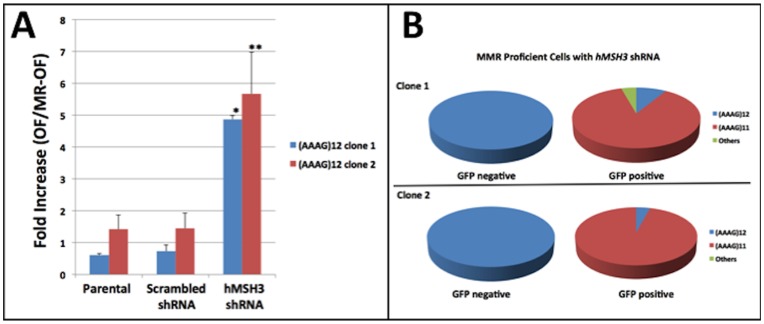
EMAST mutation frequencies in *hMSH3* knockdown CRC single-clone cells. (**A**) SW480 cells (MMR proficient) carrying the EMAST construct (D8-OF or D8-MR-OF) were transfected with *hMSH3* shRNA construct (*hMSH3* KD) or scramble control (scramble) separately. Two independent *hMSH3* and scramble KD clones as well as the parental cells were included for this study. EMAST mutation frequencies were calculated as described in Figure 1B for parental, scramble, and/or *hMSH3* KD cells. There were significantly more EGFP-positive cells in *hMSH3* KD cells compared to parent and/or scramble control cells. *: p<0.001; **: p<0.05. (**B**) EGFP-negative and -positive cells from *hMSH3* KD cells carrying D8-OF were sorted for genomic DNA isolation to examine frameshift mutations by sequencing.

Using the shRNA stably-transfected cells containing the *D8S321* locus, we examined the cell lines for occurrence of EMAST by flow cytometry. An EGFP-positive population appeared in cells where *hMSH3* expression was knocked down. *hMSH3* KD cells showed 5-fold (clone 1) and 6-fold (clone 2) increase in mutation frequencies (**Figure 3A**) compared to scrambled shRNA control cells. Reduction in hMSH3 expression greatly increased mutation rates of the *D8S321* construct to 12.7 and 18.4×10^−4^ mutation/cell/generation, compared to 1×10^−4^ mutation/cell/generation in scramble controls (**Table 2**). EGFP-negative and -positive cells were separated out for DNA sequencing to examine frameshift mutations. In both clones, greater than 90% of the EGFP negative cells retained WT sequence [(AAAG)_12_], whereas approximately 90% of the EGFP-positive cells revealed a deletion of one repeat of AAAG (**Figure 3B**). Expansion of the microsatellite repeats was also detected (included as “others” in **Figure 3B**). Overall, our observations indicate that direct KD of *hMSH3* causing *hMSH3*-deficiency alone is able to generate EMAST in previous MMR-proficient CRC cells.

**Table 2:** Calculated EMAST mutations rates for *D8S321* clones with knockdown of *hMSH3*.

	*D8S321* (AAAG)_12_clone 1	*D8S321* (AAAG)_12_clone 2
Parental	0×10^−4^±0×10^−4^	1.2×10^−4^±0.62×10^−4^
Scrambled shRNA	0×10^−4^±0×10^−4^	1.2×10^−4^±0.57×10^−4^
*hMSH3* shRNA	18.4×10^−4^±0.18×10^−4^ a	12.7×10^−4^±1.14×10^−4**a**^

Data from the EGFP-positive population at weeks 4, 5 and 6 time points were used for -4 bp frameshift mutation rate analysis. Single mutation rates were calculated by combining and averaging time-specific mutation rates. Rates are expressed as mutations at microsatellite sequence per cell per generation. Data shown are mean±SEM.

a represents significant difference in the mutation rate (*P*<0.05). *hMSH3* shRNA vs. scramble shRNA (clone 1 *P* = 0.000082, clone 2 *P* = 0.003086).

### Oxidative Stress Causes Subcellular Compartmental Shift of hMSH3 from the Nucleus to the Cytosol

**Figure 4:**
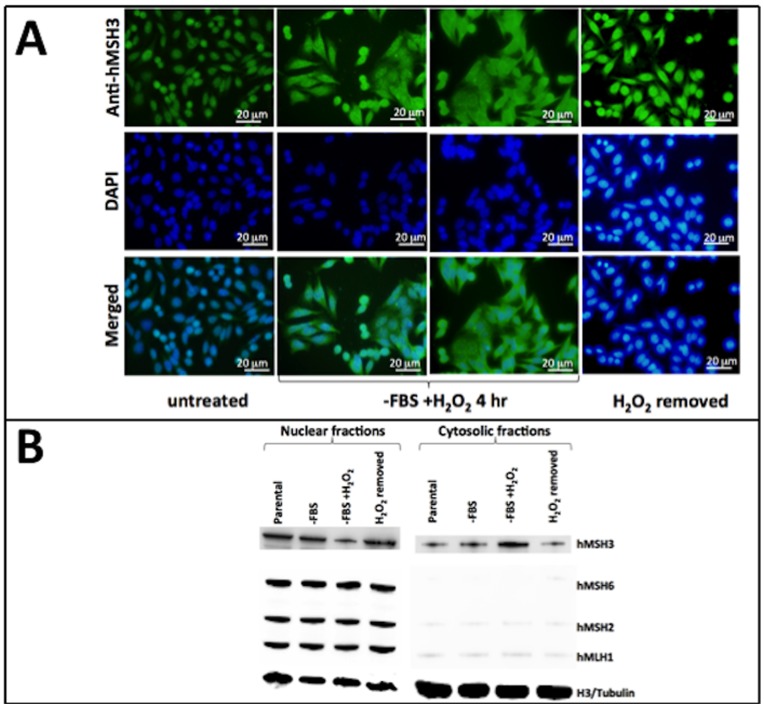
Oxidative stress causes subcellular compartmental shift from the nucleus to the cytosol for hMSH3. (**A**) MMR-proficient SW480 cells were serum-starved for 24 hours and then treated with 50 µM of H_2_O_2_ for 4 hours. After fixation, cells were stained with anti-hMSH3 antibody and then Alexa 488 conjugated anti-mouse antibody to observe the subcellular location of hMSH3. To test if the effect was reversible, cells were cultured for additional 24 hours after the removal of H_2_O_2_. (**B**) SW480 cells were serum-starved for 24 hours and then treated with H_2_O_2_ for 4 hours (−FBS+H_2_O_2_). Parental cells (Parental) and cells that were serum-starved without the subsequent H_2_O_2_ treatment (−FBS) were used as controls. Cellular proteins were separated into nuclear and cytosolic fractions using a nuclear fractionation kit. Equal volume of proteins from each group was used for SDS-PAGE and subsequent Western Blotting to check levels of hMSH3. Anti-histone H3 was used for nuclear fraction loading equality, and tubulin was used for cytosolic fraction loading equality.

EMAST has been observed to be tightly associated with heterogeneous loss of hMSH3 expression as well as nuclear heterogeneous expression (Haugen *et al*., 2008; Lee *et al*., 210, Devaraj *et al*., 2010). Our studies above further provide evidence that reduction of hMSH3 expression alone can induce EMAST in human CRC cells. We began to explore how hMSH3 might become deficient in EMAST-positive tumors with the observed reduction from full to heterogeneous expression. In CRC, EMAST is associated with inflammatory cells [13], [14], [16] that may facilitate oxidative stress, which has been shown to impair MMR function [23]. Using H_2_O_2_ as a surrogate, we investigated the effect(s) of oxidative stress on hMSH3 expression. MMR-proficient cells were treated with increasing concentrations of H_2_O_2_ (50 µM –500 µM) for up to 24 hours. We did not detect any reductions in total hMSH3 protein levels in any conditions we tried (data not shown). However, upon treating cells with 50 µM of H_2_O_2_ for 4 hours, hMSH3 was found homogenously in both the nucleus and the cytosol in a significant percentage of cells (40–60%; second panel column in **Figure 4A**). Strikingly, hMSH3 was observed to completely vacate nuclei in some cells [10–20% of total cells (third panel column in **Figure 4A**], in sharp contrast to the untreated cells where hMSH3 predominately remained localized in the nucleus (left panels in **Figure 4A**). The shift of hMSH3 localization could be detected as soon as 2 hours into treatment, and the effect peaked between 4 and 8 hours after H_2_O_2_ treatment started (data not shown). Unlike hMSH3, hMLH1, hMSH2, and hMSH6 did not alter their cellular localization and remained in nuclei (**Figure 4B** and **Figure S3A, S3B, and S3C**). Cell fractionation to separate nuclear and cytosolic fractions matched our immunocytochemistry observations (Lanes 1–3 and 5–7 in **Figure 4B**).

To examine if the effect of H_2_O_2_ on hMSH3 was reversible, we replaced H_2_O_2_ containing media with fresh media after 4 hours of treatment. hMSH3 was found to be nuclear by 24 hours after removal of H_2_O_2_ (right panels in **Figure 4A** and lanes 4 and 8 in **Figure 4B**). This result may indicate that the effect of H_2_O_2_ on hMSH3 was reversible, where hMSH3 is allowed to re-enter into the nucleus once oxidative stress abated. Alternatively, the hMSH3 that was in the cytosol might have been damaged and degraded and thus was incapable of re-entering into nuclei, and might indicate that the observed nuclear hMSH3 was newly-synthesized. To determine which scenario could be correct, we treated cells with 20 µg/ml of cyclohexamide upon removal of H_2_O_2_ to stop protein synthesis for 24 hours. hMSH3 was found to be in the nucleus (data not shown), demonstrating that the hMSH3 that was shifted to the cytosol was indeed able to re-enter into the nucleus.

## Discussion

DNA MMR studies from bacteria and yeast indicate that hMutSβ, a heterodimer of hMSH2 and hMSH3, binds and directs repair of insertion-deletion loops ±2 nucleotides [1], [5], [6]. Thus, *hMSH3*-deficiency would be expected to allow large insertion-deletion loop frameshift mutations, which has been strongly suggested in previous studies [13]–[16]. Determining the consequence of *hMSH3*-deficiency was not previously of prime importance, as there has never been described a germline mutation for *hMSH3* to cause Lynch Syndrome, and there was no correlation with sporadic CRC until the description of EMAST in CRCs [13]–[16]. The linkage of EMAST with heterogeneous loss of hMSH3 expression in sporadic CRCs suggested that this DNA MMR protein could drive EMAST. In our study, we set out to test this by creating a human cell model to measure EMAST formation in a quantitative fashion. Our study shows that (a) EMAST formation is dependent on the MMR background, with *hMSH3*-deficiency matching complete MMR-deficiency in causing EMAST, (b) *hMSH3*-deficiency mutation rates for EMAST formation are markedly higher compared to that of *hMSH6*-deficiency (essentially null for EMAST formation), (c) knockdown of *hMSH3* alone initiates formation of EMAST, and (d) oxidative stress may be one of the factors causing deficiency of hMSH3, as this MMR protein shifts its subcellular location with H_2_O_2_. Altogether, our study offers strong support to the concept that *hMSH3*-deficiency drives EMAST formation and provides a possible mechanism that may contribute to hMSH3-deficiency in human CRCs.

Tetranucleotide frameshifts that define EMAST in CRC tumors can be by insertion or deletion of tetranucleotide repeats. In our experiments, we designed constructs to detect a −4 bp deletion; however upon DNA sequencing some insertions (expansion) of the microsatellites were also detected. Indeed, the majority of frameshift mutations detected in our experiments were due to deletion of one AAAG unit (**Figure 1A** and **3B**). We selected the *D8S321* and *D20S82* loci for our experiments because deletion of one AAAG unit had been shown to be the most prevailing mutation found among loci utilized to detect EMAST [14], [16]. Because our system was designed to only detect one type of mutation, our rates calculated are likely an underestimation of the mutability of these loci with *hMSH3*-deficiency. Individual sequence context and flanking sequences may also contribute to the different outcome of the mutation (e.g. insertion vs. deletion) [11], [12], [24]. At the *D20S82* locus, only about one-third of EGFP-positive *hMSH3^−/−^* cells demonstrated one AAAG unit deletion and this could be due to this particular clone containing multiple copies of the construct. This is contrasted by more than 70% of the EGFP positive cells carrying the same construct in *hMLH1^−/−^* showing deletion of one AAAG unit, indicating the construct behaved as designed. For *hMSH3^−/−^* cells, mutation on one of the copies would lead to EGFP expression, while the rest remained WT, confounding sequencing analysis. This, however, would not affect the mutation frequency and mutation rate as the calculation was based on the number of the EGFP-positive cells.

It is not fully clear for EMAST-positive tumors how *hMSH3* is downregulated to initiate EMAST. There is clear evidence that EMAST-positive tumors are strongly associated with inflammatory cells, particularly within the epithelial components of the tumor and in the stroma surrounding the tumor (so called “tumor nests”) [16]. Thus, one hypothesis is that inflammation triggers an acquired reduction of hMSH3, which subsequently drives EMAST. Interestingly, H_2_O_2_ treatment (simulating oxidative stress) leads to a shift of hMSH3 into the cytosol away from its functional site in the nucleus without diminishing its overall expression levels. With short-term H_2_O_2_ treatment, we could not demonstrate detectable levels of EMAST formation in our model (data not shown). A longer and consistent presence of oxidative stress may be required to commence EMAST, considering EMAST appears to be associated with chronic inflammation and with advanced histology of tumors [14]. Alternatively, other components of inflammation and/or the tumor milieu may work synergistically with oxidative stress to cause EMAST. EMAST appears to be a poor prognostic factor for patients with CRC [16], [18]. This is in contrast to classic MSI for which patients with CRC have better survival at the same staged tumor of patients with MSS tumors [1]. It remains to be determined if this is intrinsic to the type of MMR defect (*hMLH1* vs. *hMSH3*) or some other factors, such as the degree or type of inflammation in the tumor.

In summary, we show through the creation of a novel cell model that *hMSH3*-deficiency drives EMAST. Our data strongly complements the observation of heterogeneous loss of hMSH3 expression in EMAST-positive tumors, and suggests that acquired *hMSH3*-deficiency, driven by oxidative stress, may be the most common DNA MMR defect among human CRCs.

## Materials and Methods

### Cell Lines and Reagents

The human colon cancer cell lines, HCT116 (*hMLH1^−/−^ hMSH3^−/−^*), DLD-1 (*hMSH6^−/−^*), HCT116+ch3 (*hMLH1-*restored but *hMSH3^−/−^*), and SW480 (MMR-proficient) cells were cultured as described previously [10]. HCT116, DLD-1, and SW480 were purchased from American Type Culture Collection (ATCC; Manassas; Virginia). HCT116+ch3 cells were kindly provided by Dr. Minoru Koi, Baylor University Medical Center, Dallas, TX [10]. Anti-hMLH1, hMSH2, hMSH3, and hMSH6 antibodies were purchased from BD Pharmigen (San Diego, CA). Anti-tubulin antibody was from Sigma (St. Louis, MO). HRP-conjugated anti-mouse antibody was purchased from Cell Signaling (Danvers, MA). Alexa 488 conjugated anti-mouse antibody was from Invitrogen (Grand Island, NY).

### Cloning of pIREShyg2-*D8S321*-EGFP and pIREShyg2-*D20S82*-EGFP Plasmids

The two human loci, *D8S321* and *D20S82,* have been reported to have the highest mutation frequency in EMAST colonic tumors [13]–[16], and were selected to construct plasmids. *D8S321* and *D20S82* sequences contain 12 and/or 16 repeats of AAAG, respectively. Sense and anti-sense oligos containing *D8S321* or *D20S82*, surrounding sequences, as well as *Pme*I and *Asc*I cloning sites (**Table S1**) were synthesized (Integrated DNA Technologies, Inc., San Diego, CA) and subjected to T4-polynucleotide kinase reactions to phosphorylate the 5′ ends (Invitrogen). The sense and anti-sense oligos were allowed to anneal and cloned into pIREShyg2-EGFP plasmids via *Pme*I-*Asc*I sites immediately after the start codon of the EGFP gene as described previously [10]–[12], [24]. Experimental plasmids were constructed +1 bp out of frame, such that one deletion of a tetranucleotide repeat within *D8S321* or *D20S82* (−4 bp frameshift mutation) would shift the reading frame into the correct frame to allow EGFP expression. Mutation resistant plasmids [MR-out-of-frame (OF)] were constructed by using sense and anti-sense oligos in which two nucleotides within every third repeat of AAAG were changed to prevent frameshift mutations. The mutation resistant in frame (MR-D8-IF) plasmid, a positive control for EGFP expression, was constructed by deleting 1 bp from *D8S321* sequence in the MR-D8-OF plasmids using Quikchange II site-directed mutagenesis kit (Stratagene, La Jolla, CA).

### *hMSH3* shRNA Construction

*hMSH3* shRNA construct and its paired empty vector were kindly provided by Dr. Ajay Goel, Baylor University Medical Center, Dallas, TX. The GFP sequence of this construct was removed by digesting the construct with *Apa*I and *Pac*I, filling-in using Klenow fragment to form blunt ends, and then performing blunt-end ligation. To make a scrambled control, the GFP sequence from the empty vector was first removed as described above and the scrambled sequence, removed from a commercially available construct (GeneCopoeia, Rockville, MD), was inserted onto the vector via *EcoR*I and *Xho*I sites. The existence and the accuracy of the scramble and/or *hMSH3* shRNA sequences on the plasmids were confirmed by DNA sequencing.

### Transfection and Generation of Stable Cell Lines

Cells were transfected with the different pIREShyg2-*D8S321*-EGFP and pIREShyg2-*D20S82*-EGFP plasmids (described in **Table S1**) by using Nucleofector kits (Amaxa, Cologne, Germany). Stable cell lines were established by hygromycin B (Invitrogen) selection. Each cell line was subjected to single cell sorting using FACS ARIA (Becton Dickinson, San Jose, CA) to establish individual clones. All single cell clones were examined by DNA sequencing to ensure the existence and accuracy of the EMAST construct they carried.

For *hMSH3* knockdown (KD) cells, MMR-proficient cells (SW480) carrying D8-OF and/or D8-MR-OF constructs were transfected with plasmids harboring scramble or *hMSH3*-specific shRNA using Fugene 6 (Roche, Mannheim, Germany), followed by puromycin (Invitrogen) selection to establish stable lines. Two independent clones from each group were used for the studies.

### Mutation Analysis by Flow Cytometry

Four hundred thousand non-fluorescent stable-transfected cells were sorted onto each well on 6-well plates by FACS ARIA (Becton Dickinson) and cultured for 4 to 6 weeks. Cells were maintained to keep them in exponential growth. To quantitate EGFP-positive populations, cells from each 100-mm dish were trypsinized, collected, and resuspended in a total volume of 2 ml of cold PBS containing 1 µg/ml of propidium iodide (PI; Sigma) right before analysis. Each group was analyzed in duplicate on a FACSCalibur (Becton Dickinson) for each time point.

For the *hMSH3* KD experiments, non-fluorescent MMR-proficient cells containing D8-MR-OF or D8-OF were sorted one week before scramble or *hMSH3*-specific shRNA transfection. Puromycin-selected scramble or *hMSH3* KD cells were analyzed by flow cytometry to quantitate EGFP-positive cells as described above.

### Polymerase Chain Reactions and DNA Sequencing

Genomic DNA from sorted EGFP-negative and/or -positive cells was PCR-amplified using a cloned *Pfu* DNA polymerase (Stratagene). The PCR products were subjected to TA cloning (Promega, Madison, WI) to obtain individual clones. Multiple clones from EGFP negative and/or positive cells were sequenced to examine the frameshift mutations. The primers used for sequencing are as following: Forward primer: 5′-GAGCTCGGATCTGTACAGGC-3′; Reverse primer: 5′TGCCGTCGTCCTTGAAGAAGA-3′.

### Determination of Frameshift Mutation Rates within *D8S321* and *D20S82* Loci

Mutation rates were calculated by the “method of the mean” developed by Luria and Delbruck [25] as described previously [10]–[12], [24].

### Western Blotting

Cells were seeded onto 6-well plates a day before protein preparation. Cells were rinsed with cold PBS and directly homogenized on the plates in cell lysis buffer (150 mM NaCl, 10 mM Tris-HCl, pH. 7.2, 0.1% IGEPAL, 0.5% Na-deoxycholate, and 5 mM EDTA) using 1 ml pipette tips. After a 15 min-incubation on ice, lysed cells were centrifuged at 10,000 rpm for 5 min at 4°C, from which clear protein lysates were collected. Protein concentration was determined using the BCA Protein Assay kit (Piece, Rockford, IL). Protein lysates were separated by 4–20% Tris-Acetate SDS-PAGE (Invitrogen) and transferred onto nitrocellulose membrane. Immunodetection was done using primary antibodies against hMLH1 (1∶1000), hMSH2 (1∶1000), hMSH3 (1∶500), hMSH6 (1∶2000), Tubulin (1∶8000), and horseradish peroxidase-conjugated secondary antibody (1∶5000). Signals were detected using Immobilon Western (Millipore) and captured by ImageQuant LAS4000 (General Electric, New York City, NY). To strip off the antibody for re-probing with a different antibody, membranes were incubated with strip-off buffer (1.5% Glycine/1% SDS/1% Tween-20, pH 2.2) at room temperature for 30 min, followed by washing/neutralizing with 1X TBST.

### Immunocytochemistry

Twenty-five thousand cells were seeded onto each well on 8-well chamber slides (Invitrogen) and incubated overnight. After serum starvation for 24 hr, cells were treated with 50 µM H_2_O_2_ for 4 hours. Cells were fixed with cold acetone on ice for 5 min, air-dried, and stored at 4°C until staining. During the staining process, slides were kept in a humidified chamber at room temperature. Slides were re-hydrated with 5% FBS/PBS (blocking/washing buffer) for 20 min before incubating with antibodies against hMLH1 (1∶100), hMSH2 (1∶100), hMSH3 (1∶100), or hMSH6 (1∶500) for 90 min and then Alexa 488 conjugated anti-mouse antibody (1∶2000) for 1 hr in dark. Slides were mounted with Prolong Gold mounting media with DAPI (Invitrogen). Pictures were taking using an Olympus DP72 Fluorescent microscope.

## Supporting Information

Figure S1 **Chromatographs for DNA sequencing of tetranucleotide microsatellites.** (**A**) The EMAST construct carrying *D8S321* marker contained 12 copies of AAAG, which underwent deletion of one copy of the AAAG unit or insertion of two copies of AAAG repeat as shown. (**B**) The *D20S82* construct harboring 16 copies of AAAG underwent deletion of one repeat of AAAG. Please note that the flanking sequences remained identical to the wild-type when EMAST markers underwent deletion/insertion.
(TIF)

Figure S2 **Expression levels of MMR proteins in clones prior to shRNA transfection.** Note that clones contained equivalent amounts of hMSH3 protein before attempted knockdown of *hMSH3*.
(TIFF)

Figure S3 **Subcellular localization of hMLH1, hMSH2, and hMSH6 upon H_2_O_2_ treatment.** After 24 hours serum-starvation and then 4 hours treatment with H_2_O_2_, cells were fixed and stained with antibodies against (**A**) hMLH1, (**B**) hMSH2, and/or (**C**) hMSH6, and then Alexa 488 conjugated anti-mouse antibody. No subcellular shift was observed for these MMR proteins.
(TIF)

Table S1 **Primer sequences (underlined) and restriction sites (** ***italicized*** **) used to insert** ***D8S321*** **and** ***D20S82*** **loci sequences onto pIREShyg2-EGFP plasmids to make the EMAST constructs.**
(TIFF)
