# Supplementary figures and images for: Oxidative Stress Induces Nuclear-to-Cytosol Shift of hMSH3, a Potential Mechanism for EMAST in Colorectal Cancer Cells
Source: PLoS One. 2012 Nov 30;7(11):e50616. doi: 10.1371/journal.pone.0050616 (PMC3511561; doi:10.1371/journal.pone.0050616)

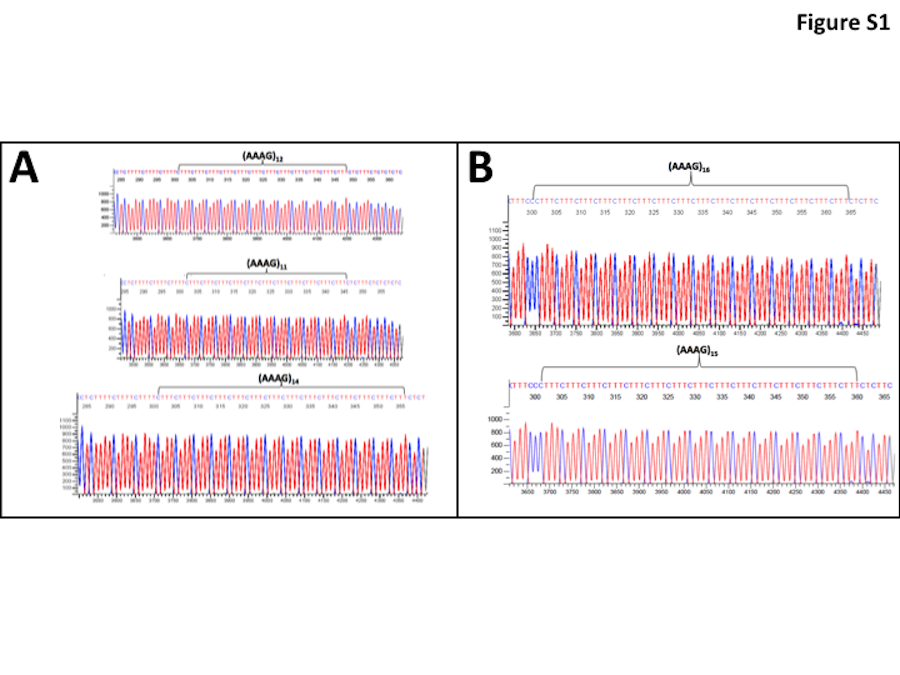

Supplement: Figure S1 — Chromatographs for DNA sequencing of tetranucleotide microsatellites. (A) The EMAST construct carrying D8S321 marker contained 12 copies of AAAG, which underwent deletion of one copy of the AAAG unit or insertion of two copies of AAAG repeat as shown. (B) The D20S82 construct harboring 16 copies of AAAG underwent deletion of one repeat of AAAG. Please note that the flanking sequences remained identical to the wild-type when EMAST markers underwent deletion/insertion. (TIF) [file pone.0050616.s001.tif]

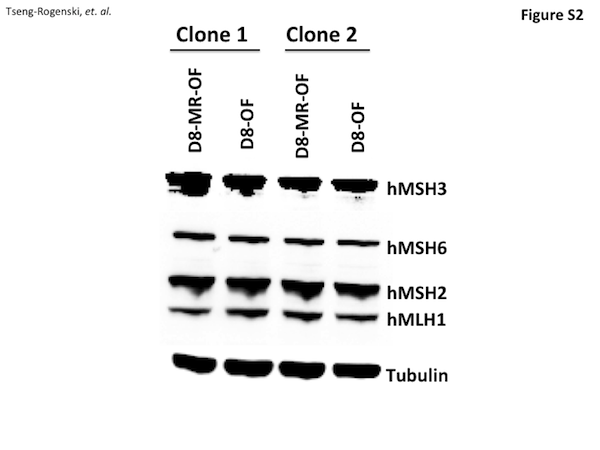

Supplement: Figure S2 — Expression levels of MMR proteins in clones prior to shRNA transfection. Note that clones contained equivalent amounts of hMSH3 protein before attempted knockdown of hMSH3. (TIFF) [file pone.0050616.s002.tiff]

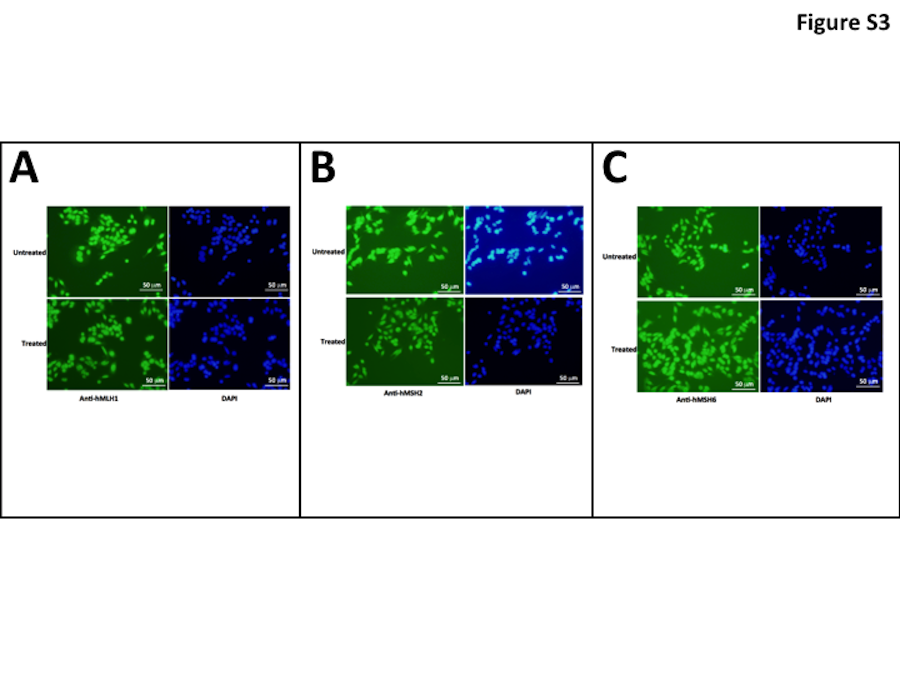

Supplement: Figure S3 — Subcellular localization of hMLH1, hMSH2, and hMSH6 upon H2O2 treatment. After 24 hours serum-starvation and then 4 hours treatment with H2O2, cells were fixed and stained with antibodies against (A) hMLH1, (B) hMSH2, and/or (C) hMSH6, and then Alexa 488 conjugated anti-mouse antibody. No subcellular shift was observed for these MMR proteins. (TIF) [file pone.0050616.s003.tif]

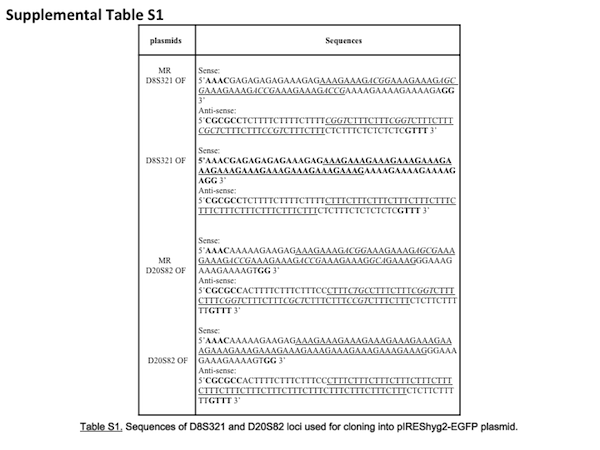

Supplement: Table S1 — Primer sequences (underlined) and restriction sites ( italicized ) used to insert D8S321 and D20S82 loci sequences onto pIREShyg2-EGFP plasmids to make the EMAST constructs. (TIFF) [file pone.0050616.s004.tiff]
